# Potential Mammalian Filovirus Reservoirs

**DOI:** 10.3201/eid1012.040346

**Published:** 2004-12

**Authors:** A. Townsend Peterson, Darin S. Carroll, James N. Mills, Karl M. Johnson

**Affiliations:** * University of Kansas, Lawrence, Kansas, USA;; † Centers for Disease Control and Prevention, Atlanta, Georgia, USA;; ‡ University of New Mexico, Albuquerque, New Mexico, USA

**Keywords:** Ebola virus, filoviridae, mammals, natural reservoir, coevolution

## Abstract

Ebola and Marburg viruses are maintained in unknown reservoir species; spillover into human populations results in occasional human cases or epidemics. We attempted to narrow the list of possibilities regarding the identity of those reservoir species. We made a series of explicit assumptions about the reservoir: it is a mammal; it supports persistent, largely asymptomatic filovirus infections; its range subsumes that of its associated filovirus; it has coevolved with the virus; it is of small body size; and it is not a species that is commensal with humans. Under these assumptions, we developed priority lists of mammal clades that coincide distributionally with filovirus outbreak distributions and compared these lists with those mammal taxa that have been tested for filovirus infection in previous epidemiologic studies. Studying the remainder of these taxa may be a fruitful avenue for pursuing the identity of natural reservoirs of filoviruses.

## Abstract 2

Biologic principles and explicit assumptions reduce the range of possibilities in identifying the reservoir of filoviruses

The virus family *Filoviridae* has been known since 1967, when Marburg virus caused an outbreak of hemorrhagic disease associated with exposure to primates imported into Germany; Marburg and Ebola viruses were subsequently the cause of isolated cases or epidemics of hemorrhagic fever in humans or nonhuman primates across Africa (*1**–**3*) and in parts of southeast Asia (*4*), and in outbreaks among nonhuman primates in North America and Europe that resulted from importation of infected primates (*5*). Despite numerous epidemiologic analyses of the disease (*6**–**8*), laboratory tests of effects of infection on potential hosts (*9*), and searches for natural virus infections among animals in localities where outbreaks have occurred (*10**–**12*), the source of these viruses in nature has remained obscure.

This article is the second step in an effort to marshal a new set of tools and approaches, designed to increase the likelihood of detecting the natural reservoirs of filoviruses. We define the reservoir that we are seeking as a set of populations or species of animal or plant that sustains the pool of virus from which infections in primates have sprung. An earlier article described the large-scale ecology and geographic distribution (Figure 1) of filovirus disease occurrences (*13*). Here, we attempt to identify the clade that constitutes the reservoir hosts of filoviruses that have caused disease in humans and in nonhuman primates. We use a series of biologic inferences regarding host-parasite interactions and make explicit assumptions to arrive at a much-reduced list of potential reservoir taxa. This approach aims to identify taxa that, under explicit assumptions, have a higher probability of constituting the reservoirs of these viruses. These lists can be used to focus future sampling and testing of potential reservoir taxa.

**Figure 1:**
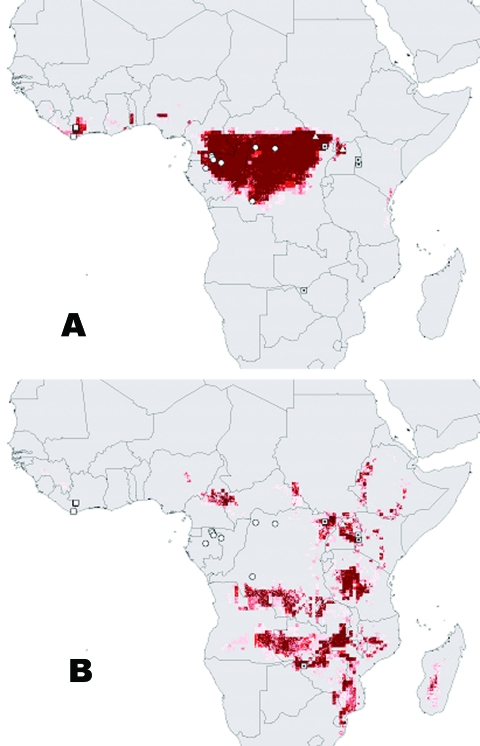
Distributional summary of A) Ebola and B) Marburg viruses, with predicted distributions based on ecologic niche models of outbreak coordinates (*13*). Darker shades of red represent increasing confidence in prediction of potential presence. Disease outbreaks attributed to various filovirus species are represented as follows: open square, Ebola Ivory Coast, open circle, Ebola Zaire, open triangle, Ebola Sudan, dotted square, Marburg.

## Rationale

The challenge of identifying the reservoirs of Ebola and Marburg viruses is complex. As previous authors (*7**,**14*) have emphasized, such a search is difficult because of the unpredictable nature of virus population dynamics in wild hosts. The challenge is also made larger because of the staggering biodiversity of tropical Africa: the array of potential hosts is immense. Subject to assumptions made explicit to the extent possible, the following rationale is an attempt to provide a scientific basis for narrowing the list of possibilities. Although any element of this list of assumptions could prove to be incorrect, the assumptions are explicit, making it possible to consider their effects.

### Mammalian Reservoir

The reservoir for filoviruses has been variously hypothesized to be a mammal, some other vertebrate, an arthropod, or even a plant (*7**,**14*). Recent evidence of a relationship between filoviruses and avian retroviruses (*15**,**16*) is intriguing, leading to the question of which major taxon is the most likely candidate for the filovirus reservoirs. No conclusive evidence based on a sufficiently broad survey exists for any of these groups as a filovirus reservoir. What little evidence exists, however, suggests that mammals may constitute an excellent first candidate for detailed consideration: 1) results of efforts to infect plants and arthropods with filoviruses have been negative (*9*); 2) small mammals (particularly bats) can sustain infections and even amplify virus (*9*); 3) certain small mammal species may have encountered filoviruses in the course of their evolutionary history (*17*); and 4) bats and other small mammals are known to serve as reservoirs for other viruses (*18*). Hence, a first assumption of this article, or a first step in the application of this approach to the challenge of detecting filovirus reservoirs, is to focus on mammals as candidate taxa.

### Persistent, Largely Asymptomatic Filovirus Infections Associated with Reservoir

Ample theoretical bases exist for the idea that in most cases a "good parasite" will evolve toward avirulence among members of a reservoir taxon with which it has a long-term evolutionary relationship (*19*), although some recent discussions suggest the contrary (*20*). Garnet and Antia (*21*) demonstrated that such coevolution results in a trade-off between host death and probability of virus transmission. Empiric evidence from studies of other viral hemorrhagic fever reservoirs provides support for the idea of evolved avirulence: the best example is the growing body of information regarding coevolution of hantaviruses and arenaviruses and their rodent hosts (*22*). In these systems, long-term associations have apparently resulted in, or at least maintained, typically asymptomatic infections of hantaviruses and arenaviruses in host rodents (*23*).

This line of reasoning suggests that the reservoir taxa will not likely include species such as the vervet monkey (*Cercopithecus aethiops*) or other primates known to experience high death rates after filovirus infections because the virus kills so quickly and efficiently that maintenance in such taxa is not likely (*7*). Hence, a second premise of this analysis is that a reservoir taxon should exist that has minimal negative effects of filovirus infection. For this reason, we eliminate primates from consideration since laboratory filovirus infection is known to result in fatal disease in both African and New World primates (*24**,**25*).

### Virus Restricted to Range of Reservoir

The range of each filovirus and the disease it causes is assumed to be restricted to the distributional range of the reservoir taxon. If the viruses exist in a natural reservoir, then their occurrence outside of the distribution of that reservoir taxon would be only fleeting, as long-term maintenance is not feasible.

### Coevolution of Filoviruses and Their Hosts

The phylogenetic structure of filovirus species has fairly clear geographic patterns (*26**,**27*). The distinct filoviruses have likely coevolved with their specific host species, implying that each virus is likely associated with a distinct host species. Therefore, we consider as unlikely reservoirs those mammal species with distributions that overlap the combined distribution of all filoviruses, although we use caution in eliminating some groups, given taxonomic arrangements in need of modern revision. However, given the preceding assumptions, reservoir species likely belong to a single genus or subfamily, with members occurring throughout the entire range of filoviruses.

Other recently described host-virus systems among small mammals have followed this pattern, especially among the hemorrhagic fever viruses. Several rodent species in the family Muridae, for example, serve as hosts for hantaviruses and arenaviruses, and patterns of cospeciation are clear when phylogenies of hosts and viruses are compared (*28**,**29*). A similar pattern may be developing for paramyxoviruses (henipaviruses), whose hosts are pteropodid bats in the southwest Pacific (*30*). We thus assume that a single, monophyletic group (genus or subfamily) of reservoir taxa associated with the filovirus lineages should exist (either for filoviruses as a whole or for the Ebola viruses and Marburg virus separately). Because the phylogenetic distance between Marburg virus and the Ebola viruses is much greater than the distance among the Ebola viruses, we provide separate reservoir candidate lists for Marburg virus, to allow for the possibility that the reservoir for Marburg virus falls within a separate reservoir taxon.

### Small Body Size of Reservoir Species

We further, if provisionally, eliminate from consideration those species of large body size. We base this assumption on two facts: large-bodied species would be eaten frequently by local people, and transmission to humans would likely be more frequent; also, contact with, or killing of, such a large animal would likely be memorable enough that either the animal would have been brought home or comment would have been made of it. For example, cases acquired by contact with infected chimps were quickly characterized (*2**,**3*). On this basis, we eliminated from consideration a variety of taxa, using an approximate cut-off of raccoon (*Procyon lotor*) size, including ungulates, Manidae, Felidae, and others.

### Reservoir Not a Commensal Species

Human filovirus infection index patients, when detailed information is available, have most frequently been men who work in the field, particularly in forests, excavations, caves, or mines (*8**,**31**,**32*). Commensal species (e.g., *Mus musculus*, *Rattus* spp.), on the other hand, might be expected to come into contact with persons working around the home. For this reason, we omit from consideration species known to be commensals with human.

## Methods

We used the following procedure and the above rationale to narrow the list of potential reservoir species for filoviruses. First, we reviewed the mammal species of the world, following the taxonomy and known distributions as summarized in Wilson and Reeder (*33*). Each species was tallied as to its approximate co-occurrence with distributions (*13*) of each filovirus (Ebola Ivory Coast in West Africa, Ebola Zaire in central Africa, and Ebola Sudan and Marburg in East Africa), as well as Ebola Reston, for which we used the entirety of the Philippines, given uncertainty as to the virus's geographic origins. Because of general geographic coincidence between Ebola Sudan and Marburg outbreaks, these two distributional areas were considered equivalent for the purpose of this first-pass, coarse-scale review.

Next, to the limits of the resolution of knowledge of phylogeny of mammals of Africa and Asia, we sought clades with geographic distributions that coincided with those of the African filoviruses. In particular, we identified genera and subfamilies with component taxa distributed in all of the filovirus distributional areas. Here, although individual species were often endemic to small areas, we detected genera or subfamilies (ostensibly monophyletic clades) with distributions that cover the entire distributional area of African filoviruses. We then noted which of these clades either also include species distributed in the Philippines, or for which related clades (i.e., same subfamily or same family) are present in the Philippines.

Finally, we reduced our genus and subfamily level lists in several ways on the basis of the assumptions outlined above. We removed clades with species known to experience high death rates from exposure to filovirus infection (e.g., primates). We removed clades with species that are frequently hunted for food or that have large body size (larger than raccoons). We removed genera for which all species are commonly commensal with humans (e.g., *Mus*). For Ebola virus, we removed species that occur exclusively in savannah habitats because Ebola disease outbreaks have occurred only in forested or ecotonal habitats (*13*); savannah-living genera were not eliminated from consideration in the lists for taxa coinciding with Marburg disease occurrences. Lists were developed for the *Filoviridae* in general, as well as for Marburg virus only; again, at the crude geographic scale of this review, owing to rough distributional coincidence between Ebola Sudan and Marburg virus outbreaks, Ebola virus distributions are more or less coincident with those of the entire family, and so the two are considered together at this point.

To assess how these lists based on coarse-scale biogeography relate to those taxa tested in epidemiologic studies to date, we reviewed all studies known to us that have involved testing of wild African mammals for filoviruses (*10**,**11**,**34**,**35*), including recent unpublished analyses (R. Swanepoel and D. Carroll, unpub. data). These lists were organized in spreadsheets, and queries were developed to establish the degree to which such studies have assessed priority taxa.

## Results

Thirty-eight genera were encountered that include species with distributions coinciding with those of all African filoviruses (Table 1). Seven of these genera, containing species of large body size, were eliminated from further consideration (*Panthera*, *Helogale*, *Herpestes*, *Aonyx*, *Genetta*, *Phacochoerus*, *Manis*). Of the remaining 31 genera, 10 either include species occurring in the Philippines or are in the same subfamily as genera occurring in the Philippines.

**Table 1:** Genera for which component species coincide distributionally (coarse-scale) with distributions of disease outbreaks associated with all four known African filoviruses^a^

Genus^b^	Species
**Insectivora: Soricidae (shrews)**	
*Sylvisorex+*	*johnstoni*, *megalura*
*Crocidura* ^c^	**Many species**
*Suncus* ^c^	*etruscus, infinitesimus, lixus, remyi, varilla*
**Chiroptera: Pteropodidae (fruit bats)**	
*Epomophorus+*	***labiatus***, *minimus*
*Epomops+*	*buettikoferi*, *dobsoni*, ***franqueti***
*Rousettus* ^c^	*aegyptiacus*, *angolensis*, *lanosus*
**Chiroptera: Emballonuridae (sac-winged bats)**	
*Taphozous* ^c^	*hamiltoni*, *hildegardeae*, ***mauritianus***, *nudiventris*
**Chiroptera: Nycteridae (slit-faced bats)**	
*Nycteris–*	*arge*, *gambiensis*, *grandis*, ***hispida***, *intermedia*, *macrotis*, *major*, *nana*
**Chiroptera: Rhinolophidae (horseshoe bats)**	
*Rhinolophus* ^c^	*adami*, *alcyone*, *deckenii*, *denti*, *eloquens*, *fumigatus*, *guineensis*, *hildebrandti*, *landeri*, *maclaudi*, *silvestris*, *simulator*
*Hipposideros* ^c^	*beatus*, *caffer*, *camerunensis*, *commersoni*, ***cyclops***, *fuliginosus*, *jonesi*, *lamottei*, *marisae*, ***ruber***
**Chiroptera: Vespertilionidae (vesper bats)**	
*Kerivoula* ^c^	*argentata*, *cuprosa*, *lanosa*, *phalaena*
***Chalinolobus+***	*alboguttatus*, *argentatus*, *beatrix*, *egeria*, *gleni*, *poensis*, *superbus*, *variegatus*
*Eptesicus+*	*brunneus*, *capensis*, *guineensis*, *hottentotus*, *platyops*, *rendalli*, ***somalicus***, ***tenuipinnis***
*Myotis* ^c^	***bocagei***, *tricolor*
*Pipistrellus* ^c^	*crassulus*, *eisentrauti*, *inexspectatus*, *musciculus*, *nanulus*, ***nanus***, *rusticus*
*Scotoecus+*	*albofuscus*, ***hirundo***
*Scotophilus* ^c^	***dinganii***, *nigrita*, *nux*, *robustus*, *viridis*
*Miniopterus* ^c^	*inflatus*, ***minor***, *schreibersi*
**Chiroptera: Molossidae (free-tailed bats)**	
*Chaerephon* ^c^	*aloysiisabaudiae*, ***ansorgei***, *bemmeleni*, *bivittata*, *chapini*, *gallagheri*, ***major***, *nigeriae*, ***pumila***, *russata*
*Mops+*	*brachypterus*, ***condylurus***, ***congicus***, *demonstrator*, *midas*, ***nanulus***, *niangarae*, ***niveiventer***, *spurrelli*, ***thersites***, ***trevori***
*Myopterus+*	*daubentonii*, ***whitleyi***
*Tadarida+*	*aegyptiaca*, *fulminans*
**Rodentia: Sciuridae (squirrels)**	
*Funisciurus+*	***anerythrus***, *bayonii*, *carruthersi*, ***congicus***, ***isabella***, ***lemniscatus***, *leucogenys*, ***pyrropus***, *substriatus*
*Heliosciurus+*	*gambianus*, *mutabilis*, *punctatus*, ***rufobrachium***, *ruwenzorii*, *undulatus*
*Protoxerus+*	*aubinnii*, ***stangeri***
**Rodentia: Muridae (mice and rats)**	
*Dendromus–*	*insignis*, *kahuziensis*, *kivu*, *melanotis*, ***mesomelas***, *messorius*, ***mystacalis***, *nyikae*
*Grammomys+*	***aridulus***, *buntingi*, *caniceps*, ***dolichurus***, *dryas*, *gigas*, *ibeanus*, *macmillani*, ***rutilans***
*Lemniscomys+*	*barbarus*, *bellieri*, *hoogstraali*, *linulus*, *macculus*, *rosalia*, ***striatus***
*Lophuromys+*	*cinereus*, ***flavopunctatus***, *luteogaster*, *medicaudatus*, *nudicaudus*, *rahmi*, ***sikapusi***, *woosnami*
*Malacomys+*	*cansdalei*, *edwardsi*, *longipes*, *lukolelae*, *verschureni*
**Rodentia: Anomaluridae (scaly-tailed squirrels)**	
*Anomalurus*	***beecrofti***, ***derbianus***, *pelii*, *pusillus*
**Rodentia: Myoxidae (dormice)**	
***Graphiurus***	*christyi*, *crassicaudatus*, *hueti*, *lorraineus*, *microtis*, *murinus*, *parvus*

^a^Component African species are listed (only those coinciding with one or more specific filovirus distributional areas); species that have been tested in epidemiologic studies are indicated in **boldface**. ^b^Genera for which related genera occur in the Philippines are indicated by +, for same subfamily, and –, same family. ^c^Genera including species occurring in the Philippines.

Considering coincidence of generic distributions with only Marburg hemorrhagic fever occurrences (Table 2), an initial list included 63 genera; 22 of these were omitted because their species had a large body size or were primates (*Perodicticus*, *Galago*, *Gorilla*, *Leptailurus*, *Atilax*, *Dologale*, *Mungos*, *Crocuta*, *Lutra*, *Civettictis*, *Ceratotherium*, *Orycteropus*, *Potamochoerus*, *Litocranius*, *Taurotragus*, *Tragelaphus*, *Cephalophus*, *Sylvicapra*, *Oryx*, *Kobus*, *Redunca*, *Manis*). Of the remaining 41 genera, 3 include species occurring in the Philippines, 18 have consubfamilials occurring in the Philippines, and 29 have confamilials occurring in the Philippines.

**Table 2:** Genera that coincide distributionally with the geographic distribution of disease outbreaks associated with Marburg virus^a^

Genera^b,c^	Species coinciding with Marburg virus
**Insectivora: Chrysochloridae (golden moles)**	
*Chrysochloris*	*stuhlmanni*
**Insectivora: Soricidae (shrews)**	
*Suncus^b^*	*etruscus, infinitesimus, lixus, varilla*
**Chiroptera: Pteropodidae (fruit bats)**	
*Epomops–*	***franqueti*** *, dobsoni*
*Epomophorus–*	*gambianus, grandis, crypturus, * ***labiatus*** *, minimus, wahlbergi*
**Chiroptera: Emballonuridae (sac-winged bats)**	
*Saccolaimus* ^b^	***peli***
**Chiroptera: Megadermatidae (false vampire bats)**	
*Lavia+*	*frons*
**Chiroptera: Rhinolophidae (horseshoe bats)**	
*Cloeotis+*	*percivali*
**Chiroptera: Vespertilionidae (vesper bats)**	
*Mimetillus+*	*moloneyi*
**Rodentia: Sciuridae (squirrels)**	
*Xerus+*	*erythropus*, *inauris*, *rutilus*
**Rodentia: Muridae (mice and rats)**	
*Acomys+*	*kempi*, *spinosissimus*, *ignitus*, *percivali*, *wilsoni*
***Aethomys+***	*namaquensis*, *kaiseri*, *hindei*, *chrysophilus*
*Arvicanthis+*	*niloticus*, *nairobae*
*Beamys–*	*hindei*, *major*
*Cricetomys–*	***gambianus***
*Dasymys+*	***incomtus***
*Delanymys–*	*brooksi*
*Deomys–*	***ferrugineus***
*Gerbillurus–*	*paeba*
*Hybomys+*	***univittatus***, *lunaris*
*Hylomyscus+*	***stella***, *denniae*
*Lophiomys–*	*imhausi*
***Mastomys***	*erythroleucus*, *pernanus*, *hildebrandtii*
*Mylomys+*	*dybowskii*
*Oenomys+*	***hypoxanthus***
*Otomys–*	*anchietae*, *typus*, *tropicalis*, *angoniensis*, *denti*
*Praomys+*	*misonnei*, ***jacksoni***
*Rhabdomys+*	*pumilio*
*Steatomys–*	*parvus*, ***pratensis***
***Stochomys+***	*longicaudatus*
*Tachyoryctes–*	*naivashae*, *annectens*, *ankoliae*, *spalacinus*, *ruddi*, *ruandae*
*Tatera–*	*robusta*, *leucogaster*, *inclusa*, *boehmi*, *nigricauda*
*Taterillus–*	*harringtoni*, *emini*
*Thallomys+*	*loringi*, *paedulcus*
*Uranomys+*	*ruddi*
*Zelotomys+*	*hildegardeae*
**Rodentia: Anomaluridae (Scaly-tailed Flying Squirrels)**	
*Idiurus*	*zenkeri*
**Rodentia: Pedetidae (springhare)**	
*Pedetes*	*capensis*
**Rodentia: Thryonomyidae (cane rats)**	
*Thryonomys*	*swinderianus*, *gregorianus*
**Lagomorpha: Leporidae (rabbits and hares)**	
*Poelagus*	*marjorita*
**Macroscelidea: Macroscelididae (elephant shrews)**	
*Petrodromus*	***tetradactylus***
*Rhynchocyon*	*chrysopygus*, ***cirnei***, *petersi*

^a^Genera that have been tested in epidemiologic studies are indicated in **boldface**. ^b^Genera, including species occurring in the Philippines. ^c^Genera for which related genera occur in the Philippines are indicated by +, same subfamily; or –,same family.

Finally, we considered clades recognized at the subfamilial (or familial, if no subfamilies were recognized; Table 3) level that coincided with all African filovirus occurrences or Marburg occurrences only. Of these 65 clades, we eliminated 33 because their members had large body size or were primates (Loridae, Galagonidae, Cercopithecinae, Colobinae, Hominidae, Canidae, Acinonychinae, Felinae, Pantherinae, Herpestinae, Hyaeninae, Protelinae, Lutrinae, Mellivorinae, Mustelinae, Nandiniinae, Viverrinae, Elephantidae, Rhinocerotidae, Orycteropodidae, Aepycerotinae, Alcelaphinae, Antilopinae, Bovinae, Cephalophinae, Hippotraginae, Reduncinae, Phacochoerinae, Suinae, Hippopotamidae, Tragulidae, Giraffidae, Manidae). Of the 32 remaining subfamilies, 8 coincided with Marburg virus occurrences only, and 11 with all African filovirus or all African Ebola virus disease occurrences; 13 not only coincided with African filovirus disease occurrences but also included distributional areas in the Philippines (Table 3).

**Table 3:** Subfamilial- or familial-level taxa that hold genera with distributions that coincide (coarse-scale) with distributions of disease outbreaks associated with African filoviruses^a^

Subfamily or family
Coincides with Marburg occurrences only
**Chrysochloridae**
Otomyinae
Petromyscinae
Rhizomyinae
Lophiomyinae
Pedetidae
**Bathyergidae**
Macroscelididae
Coincides with African filovirus occurrences
**Crocidurinae** ^b^
Potamogalinae
**Pteropodinae** ^b^
**Macroglossinae** ^b^
**Emballonuridae** ^b^
Megadermatidae^b^
Rhinolophinae^b^
**Hipposiderinae** ^b^
Kerivoulinae^b^
**Vespertilioninae** ^b^
**Miniopterinae** ^b^
**Molossidae** ^b^
**Nycteridae**
Procaviidae
**Sciurinae** ^b^
**Murinae** ^b^
**Cricetomyinae**
**Dendromurinae**
**Gerbillinae**
**Anomalurinae**
Zenkerellinae
**Graphiurinae**
**Thryonomyidae**
Leporidae

^a^Taxa including species that have been tested are indicated in boldface. ^b^Genera including species occurring in the Philippines.

Of the 134 species that have been tested in previous studies (*11**,**12**,**34**,**35*) (R. Swanepoel and D. Carroll, unpub. data), only 58 are from genera that coincide with African filovirus disease occurrences. Overall, of 4,709 mammals tested, only 2,545 were from clades with distributions coincident at some taxonomic level with that of African filovirus disease outbreaks.

**Figure 2:**
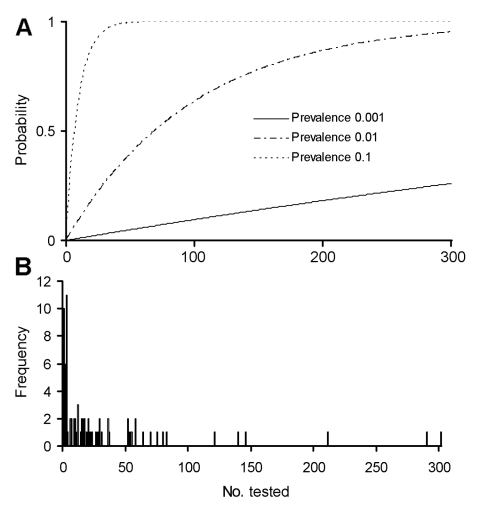
Summary of numbers per species that have been tested in studies seeking filovirus infections in wild mammals (Table A1). A) Theoretical probabilities of detecting the reservoir in samples of particular sizes, given prevalences of 0.1%, 1%, and 10%. B) Frequency (1–11 species) with which species have been tested for filoviruses.

Of the taxa that geographically cooccur with filovirus disease occurrences (Table 1, Table 2, Table 3), variable numbers have been tested (Table A1): genera coinciding with filovirus disease occurrences, 26 (81.2%) of 32; genera coinciding with Marburg, 14 (40.0%) of 35; subfamilies coinciding with filovirus occurrences, 17 (70.8%) of 24; and subfamilies coinciding with Marburg occurrences, 2 (25%) of 8. Hence, considering the lists presented in Table 1, Table 2, and Table 3, a significant diversity of taxa remains to be tested even a single time. If prevalences are anything other than high, testing greater numbers of mammals from some clades will also be necessary (Figure 2); the list of clades not tested satisfactorily thus becomes quite long.

## Discussion

Searches for the filovirus reservoirs have been conducted periodically since filovirus diseases were first recognized. Nevertheless, until the present, very little practical or theoretical information was available to help researchers plan trapping expeditions, choose sites and trapping techniques, or focus on particular species. This analysis should help in these aspects of planning and may reduce the number of hours that researchers spend sampling low-priority habitats or species.

The preliminary, coarse-filter analyses presented here rely on a series of explicit assumptions drawn from past studies of filovirus disease outbreaks and from biologic principles and theory. Regarding the first two assumptions—that reservoirs exist and that they are mammals—filovirus disease outbreaks could conceivably be maintained through rapid and efficient transmission among highly susceptible taxa such as primates. No historic evidence for this hypothesis has been assembled, but a recent epidemic among nonhuman primates in Gabon and the Democratic Republic of the Congo has been intense and continuous; some believe this outbreak may be being transmitted among primate populations and may be pushing chimpanzee and gorilla populations towards extinction (*36**,**37*).

That the reservoir is a mammal is a more tenuous assumption. Numerous suggestions have been made regarding potential nonmammal reservoirs, including arthropods and plants, but no experimental evidence has documented either successful infection of these taxa or long-term infection that suggests maintenance of virus in the host (*9*). Although many possible alternatives exist, given successful laboratory infections of several mammal taxa, this clade seems a logical starting point. These procedures are feasibly applicable only to taxa that are reasonably well-known, both taxonomically and distributionally, such as mammals and birds. Recent analyses of filovirus nucleic acids, however, suggest that "filoviruses are more closely related to paramyxoviruses, particularly human respiratory syncytial virus" (*15*). As such, other vertebrates that host paramyxoviruses—birds, reptiles, and amphibians—may also merit study (*15**,**16*).

The assumption that the reservoir should be relatively nonsusceptible to negative effects of filovirus infection is based in large part on theory regarding host-parasite coevolution (*19**,**20*). A reservoir that is coevolved with the virus and experiences less severe effects of infection would lend greater long-term stability and lower probability of extinction to the pathogen populations.

The assumption of distributional coincidence between reservoir and virus is key to our inferences. Viruses not uncommonly escape from their natural reservoirs and are maintained at least temporarily in an alternate host (e.g., Nipah virus, influenza viruses, West Nile virus, even Ebola viruses in primate populations). However, these events do not go unnoticed, and we assume that such an escape from natural maintenance would be detected, as it has been in the past. That filoviruses could not persist long-term without the coevolved reservoir taxon's being present is fairly clear. This effect should ensure that virus distributions will be coincident with or nested within host distributions. The converse, however, is not necessarily clear: reservoir taxa can exist in areas in which the virus is not present (*18*).

Our assumption that the reservoir would be a species with a small body size is perhaps the most tenuous. However, we suspect that large-sized species would be hunted more often, which would provide more frequent opportunities for human infections. Also, contact with larger-sized species would be more notable and would likely be mentioned to family members before symptoms appeared. Finally, phylogenetic patterns among filoviruses suggest cospeciation within a similarly diverse clade (probably a genus or subfamily) of host species, and highly diverse genera or species complexes are less common among larger bodied mammals.

The clades identified in our analyses represent a broad swath of African mammal diversity. Dominant are bats, rodents, and insectivores. When Ebola or all African filovirus outbreaks are considered, bat clades dominate the lists, whereas when only Marburg outbreaks are considered, rodents enter the picture more broadly, reflecting the greater diversity of rodents in the arid habitats that characterize the known distributional area of Marburg virus.

Where do these explorations take us? We suggest four important adjustments to the epidemiologic studies that accompany most filovirus disease outbreaks. First, attention should focus on species that are spatially coincident with the aggregate distribution of outbreaks attributed to a given filovirus and that are from clades coincident with the distribution of filoviruses in general. Second, rather than testing species that are most common proportionally, collectors should attempt to sample each species and clade that co-occurs with known ranges of filovirus disease outbreaks, particularly given the rarity of reservoir-to-human transmission events. Third, emphasis should be placed on testing samples of each species large enough to give a reasonable probability of actual detection. For example, if the prevalence of filoviruses in the reservoir were 1%, then a sample of ≈60 to 70 would provide only a 50% chance of detecting the virus, and a sample of >200 would be needed to have a 90% chance of detection (Figure 2) (*38*). Fourth, publication of negative results in testing for filovirus infections will be important in guiding future studies and making them still more efficient. These recommendations imply the need to bring specialized expertise on mammals to bear on the collecting challenge. Capturing animals of some taxa will require specialized equipment and techniques, as well as carefully planned strategies. Knowledge of the natural history and ecology of each of the key taxa will be important to successful sampling. Enlisting the assistance of local hunters and trappers who have specific experience with native species has proven very helpful in previous investigations (J. Mills and D. Carroll, unpub. data). We believe that concentrating sampling efforts on the taxa listed in Table 1, Table 2, and Table 3 will improve the chances of discovering filovirus reservoir species.

Laboratory inoculation studies also should focus on candidate taxa identified herein and should attempt to identify species that support persistent and largely asymptomatic infections with filoviruses. Such studies will serve to further direct field sampling efforts toward the most likely reservoir candidates. Investigators should be cognizant of new regulations regarding the importation of African rodents or rodent tissues and obtain all required permits (*39*).

In summary, our aim has been to use a series of biologic principles to guide reasoning towards narrowing the list of potential mammal reservoirs for filoviruses. These lists can guide sampling efforts, even when disease outbreaks are not ongoing. We do not imply certainty that the reservoir is among the species on our lists. We do, however, suggest that, under explicit assumptions, we have identified a suite of clades with a higher probability of being filovirus reservoir hosts than remaining African mammal clades. These lists can serve as a guide to future mammal surveys, allowing investigators to focus sampling efforts on high-probability taxa. In future studies, we will apply tools from ecologic niche modeling to refine and reduce these lists still further.

## Appendix

**Table A1:** Species tested in past epidemiologic studies^a^ for filovirus infections, with numbers of individual species tested^b^

Species	Priority^b^	Arata and Johnson 1978	Germain 1978	Leirs et al. 1999	Breman 1999	CDC/NIV/CIRMF 2002	Total

Insectivora: Chrysochloridae (golden moles)							
*Chlorotalpa leucorhina*				5			5
Insectivora: Soricidae (shrews)							
*Crocidura attila?*	G			1			1
*C. denti*	G			3			3
*C. dolichura?*	M			1			1
*C. flavescens*	G	7				10	17
*C. hildegardeae?*	G			3			3
*C. hirta*	M			12			12
*C. littoralis?*	M			3			3
*C. olivieri*	G			55			55
*C. parvipes*	G			3			3
*C. poensis* group	G			20	9		29
*Crocidura* sp.	G			5		6	11
*Scutisorex somereni*					6		6
Chiroptera: Pteropodidae (fruit bats)							
*Casinycteris argynnis*				2			2
*Eidolon helvum*	S				6		6
*Epomophorus gambiensis*						1	1
*E. labiatus*		2					2
*Epomops franqueti*	SG			2	21	6	29
*Epomops* sp.	G					3	3
*Hypsignathus monstrosus*	S		1			30	31
*Micropteropus pusillus*	S	2		78			80
*Myonycteris torquata*	S					2	2
*Rousettus* sp.						39	39
*Scotonycteris* sp.	G					3	3
*Megaloglossus woermanni*	S			43		9	52
Chiroptera: Emballonuridae (sac-winged bats)							
*Saccolaimus peli*	S				9		9
*Taphozous mauritanius*	SG	12					12
Chiroptera: Nycteridae (slit-faced bats)							
*Nycteris hispida*	SG			2			2
*Nycteris* sp.	G				14		14
Chiroptera: Rhinolophidae (horseshoe bats)							
*Hipposideros cyclops*	SG				52	1	53
*H. ruber*	SG				17		17
Chiroptera: Vespertilionidae (vesper bats)							
*Chalinolobus* sp.	G				15		15
*Eptesicus somalicus*	SG			1			1
*E. tenuipinnis*	SG			1	22		23
*Miniopterus minor*	MG			2			2
*Myotis bocagei*	SG			2	17		19
*Pipistrellus nanus*	SG			2	73		75
*Scotoecus hindei*		2					2
*Scotophilus dinganii*	SG			20			20
*S. hirundo*	SG	12					12
*Scotophilus* sp.	G				10		10
Chiroptera: Molossidae (free-tailed bats)							
*Chaerephon ansorgei*	MG			121			121
*C. major*	MG				26		26
*C. pumila*	SG			211			211
*Mops condylurus*	SG			10	54		64
*M. congicus*	SG				20		20
*M. nanulus*	SG	8		14	15		37
*M. niveiventer*	G			3			3
*M. thersites*	SG			1	69		70
*M. trevori*	MG	140					140
*Myopterus whitleyi*	SG			2			2
Primata: Loridae (Lorises)							
*Perodicticus potto*					8		8
Primata: Galagonidae (galagos and bushbabies)							
*Galago alleni*						1	1
Primata: Cercopithecidae (Old World Monkeys)							
*Allenopithecus nigroviridis*					10		10
*Cercocebus galeritus*					11		11
*Cercopithecus ascanius*			2	4	94		100
*C. neglectus*			1		12		13
*C. nictitans*			1	1	51		53
*C. pogonias*			1		55		56
*Procolobus pennantii*					14		14
Carnivora: Canidae (dogs and wolves)							
*Canis aureus*				1			1
*C. domesticus*					14		14
*C. mesomelas*				1			1
Carnivora: Herpestidae (mongooses)							
*Crossarchus alexandri*					7		7
*Herpestes sanguineus*				3			3
*Mungos mungo*				1			1
Carnivora: Viverridae (civets, genets, linsangs, and relatives)							
*Genetta servalina*				3			3
*G. tigrina*				3			3
*Nandinia binotata*				16			16
Artiodactyla: Bovidae (antelopes, gazelles, cattle, sheep, goats)							
*Cephalophus monticola*			2	9	17		28
*Neotragus pygmaeus*					6		6
*Sylvicapra grimmia*				4			4
*Tragelaphus scriptus*				1			1
Pholidota: Manidae (pangolins)							
*Manis tricuspis*				29	66		95
Rodentia: Sciuridae (squirrels)							
*Epixerus ebii*						2	2
*Funisciurus anerythrus*	MG				58		58
*F. congicus*	G			36			36
*F. isabella*	G					2	2
*F. lemniscatus*	G				6	1	7
*F. pyrrhopus*	SG			15			15
*Funisciurus* sp.	G		2				2
*Heliosciurus rufobrachium*	SG				58		58
*Paraxerus alexandri*	M			1			1
*P.* sp.					6		6
*Protoxerus stangeri*	SG			1		2	3
Rodentia: Muridae (mice and rats)							
*Cricetomys emini*					19		19
*C. gambianus*	M	2		5			7
*Dendromus mesomelas?*	MG			52			52
*D. mystacalis?*	MG			3			3
*Dendromus* sp.	G			4			4
*Deomys ferrugineus*	M					3	3
*Steatomys pratensis*	M			16			16
*Tatera valida*				20			20
*T.* sp.				3			3
*Aethomys* sp.		4					4
*Colomys goslingi*				1			1
*Dasymys incomtus*	M			6			6
*Grammomys avidulus*	G	3					3
*G. dolichurus*	G			9			9
*G. rutilans*	SG			1			1
*Heimyscus fumosus*						3	3
*Hybomys univittatus*					50	2	52
*Hylomyscus carillus*				14			14
*H. parvus*						1	1
*H. stella*	M				11	5	16
*Hylomyscus* sp.			1			5	6
*Lemniscomys striatus*	SG	10	1	249	30		290
*Lophuromys flavopunctatus*	MG			1			1
*L. sikapusi*	SG	6	1	58	81		146
*Malacomys lukolelae*	G			36			36
*Mastomys natalensis*		180		62			242
*Mastomys* sp.					72		72
*Mus minutoides*				289		2	291
*M. triton*				459			459
*Oenomys hypoxanthus*	M			1	82		83
*Pelomys campanae*				32			32
*P. minor*				21			21
*Pelomys* sp.				7			7
*Praomys jacksoni*	M			241	60		301
*P. tullbergi*			69		7	2	78
*Praomys* sp.						7	7
*Rattus rattus*		103	30	89			222
*Rattus* sp.					52		52
*Stochomys* sp.					8		8
*Thamnomys rutilans*			5		13		18
Rodentia: Anomaluridae (Scaly-tailed Flying Squirrels)							
*Anomalurus beecrofti*	SG					1	1
*A. derbianus*	MG			1			1
Rodentia: Myoxidae (dormice)							
*Graphiurus lorraineus*	SG			1			1
*G.* sp.	G		3		19		22
Rodentia: Bathyergidae (mole rats and blesmoles)							
*Cryptomys ochraceocinereus*		1					1
Rodentia: Hystricidae (Old World Porcupines)							
*Atherurus africanus*	SG				10		10
Rodentia: Thryonomyidae (cane rats)							
*Thryonomys swinderianus*				18			18
Macroscelidea: Macroscelididae (elephant shrews)							
*Petrodromus tetradactylus*	M			1			1
*Rhynchocyon cirnei*	M				28		28
Total		494	120	2,456	1,490	149	4,709

^a^ (*40**–**43*), R. Swanepoel and D. Carroll, unpub. data. ^b^Priority refers to presence on the lists in preceding tables: S = species coinciding with all African filovirus or all African Ebola virus occurrences, M = species coinciding with all Marburg occurrences, G = genera coinciding with all African filovirus or all African Ebolavirus occurrences.
